# Associations between english proficiency and HPV vaccine uptake among foreign-born men and women in the United States: a cross-sectional study using data from the National Health Interview Survey

**DOI:** 10.1186/s12889-025-25555-2

**Published:** 2025-11-28

**Authors:** Nubwa St. James, Lois Coleman Carpenter, James O. Nyamao, Dan Li, Catherine S. Nagawa

**Affiliations:** 1 Department of Health Promotion Sciences, University of Oklahoma Health Science Center, Oklahoma City, OK USA; 2 TSET Health Promotion Research Center, Stephenson Cancer Center, University of Oklahoma Health Science Center, Tulsa, OK USA

**Keywords:** HPV, English proficiency, Foreign-born, HPV vaccine, Human papillomavirus

## Abstract

**Background:**

Disparities in human papillomavirus (HPV) vaccination persist among foreign-born adults in the U.S., who are up to 30% less likely than their U.S.-born counterparts to have ever received the vaccine. While HPV vaccination primarily targets adolescents, the recent expansion of eligibility to adults provides an opportunity to reach individuals who missed vaccination during adolescence. However, English often serves as a second or third language for many foreign-born adults, and limited proficiency or dialect differences may create barriers to navigating the U.S. healthcare system and accessing preventive services. Using data from the National Health Interview Survey, this study examines whether self-reported English proficiency is associated with HPV vaccine uptake among foreign-born U.S adults.

**Methods:**

We analyzed data from the 2015–2018 National Health Interview Survey (NHIS). Participants were foreign-born adults aged 18–38 years for women, and 18–36 years for men at the time of data collection (*n* = 5,450). The dependent variable was self-reported ever receipt of an HPV vaccine (yes/no), and the independent variable was English proficiency, dichotomized as high vs. low English proficiency. We used logistic regression to estimate odds ratios (ORs) and 95% confidence intervals (95% CIs) for the association between English proficiency and receipt of the HPV vaccine. All estimates were weighted to account for the NHIS’s complex survey design.

**Results:**

The weighted prevalence of ever receiving the HPV vaccine was 12.2% (95% CI: 11.1–13.3) among foreign-born adults in the U.S. The overall mean age was 29.4 years (95% CI: 29.2–29.6). Among those who had received the HPV vaccine, 76.7% were female and 23.3% were male, whereas among those who had not received the vaccine, 49.9% were female and 50.1% were male (*p* < 0.001). We found that foreign-born individuals with low English proficiency were significantly less likely to have received the HPV vaccine than those with high proficiency (OR: 0.48; 95% CI: 0.32–0.73) after adjusting for sex, age, marital status, citizenship status, education, geographical region of birth, insurance status, and having a usual source of care.

**Conclusion:**

Our findings suggest that limited English proficiency negatively influences the receipt of HPV vaccination among foreign-born individuals in the U.S. Targeted interventions for populations in need of linguistically tailored education and interactions within healthcare systems are needed to address disparities in HPV vaccination.

## Introduction

 The human papillomavirus (HPV) infection is the most common sexually transmitted infection globally, accounting for 5% of the cancer burden, including cervical, oropharyngeal, anal, penile, vaginal, and vulvar cancer [1]. Globally, HPV infections contributed to an estimated 800,000 new cases and 420,000 deaths across several cancer types in 2022 [2]. The incidence rate of HPV-related cancers varies by country’s economic status, with age-standardized incidence rates ranging from 5.2 to 13.5 per 100,000 people in regions such as North Africa, Western Asia, and Southern Europe, and 27.4–72.4 per 100,000 people in Africa, Southern Asia, and South America [2]. These variations are attributable to the differences in access to and use of the HPV vaccine, as well as cervical cancer screening and treatment across regions [3]. Regions with widespread access and use of the vaccine have seen substantial declines in vaccine-type HPV genital infections and cervical cancers, a gain not yet achieved by regions with limited access [4].

In the U.S, HPV infection is linked to over 40,000 cancer cases annually [5]. Close to 4% of women in the U.S are estimated to have high-risk cervical HPV infections at any given time [6]. The HPV vaccine was first licensed by the U.S. Federal Drug Administration in 2006 for use in females aged 9–26 years [7]. In 2009, following clinical trial evidence demonstrating the vaccine’s effectiveness in preventing HPV-related diseases, including genital warts and precancerous lesions, recommendations were expanded to include males of similar ages, making the U.S. the first country to do so [7, 8]. Early uptake patterns revealed significant disparities that have persisted over time [9–11]. Although HPV vaccination coverage in the U.S. is moderately high at 61%, substantial disparities remain, with foreign-born populations up to 30% less likely than their U.S.-born counterparts to have received the vaccine [12–15]. Reasons for the relatively low HPV vaccination uptake in foreign-born individuals may be driven by factors impacting access to the vaccine in their countries of birth and further compounded by social determinants of health in the U.S., including cost, insurance status, and language barriers [16–23]. HPV vaccines are a highly effective primary strategy for preventing HPV infections and HPV-related cancers, such as cervical and oropharyngeal cancer [24]. Therefore, increasing the uptake of the vaccine remains relevant to public health.

While HPV vaccination recommendations have primarily targeted adolescents and young adults aged 9–26 years, recent expansions to include adults aged 27–45 years provide an important opportunity for individuals who missed the recommended vaccination window to catch up on the vaccine [25]. Adult catch-up vaccination is critical for reducing the burden of persistent HPV infections, preventing HPV-related cancers among those not vaccinated earlier, and closing population-level coverage gaps [25]. This is particularly important for foreign-born adults, who are less likely to be vaccinated and could benefit from the expanded recommendations to gain protection against HPV and related cancers.

English is a second or later language for some individuals born outside the U.S. English proficiency (i.e., the ability to communicate effectively in the English language) [26], and dialect differences may present challenges for foreign-born individuals when navigating the U.S. healthcare system, impeding access to healthcare in this group [17, 27]. Language barriers related to English proficiency may therefore negatively correlate with patient-provider communication, limit understanding of medical terminology, and impede appointment scheduling over the phone [28–32]. Despite the significance of English proficiency to the uptake of cancer preventive services and healthcare access among immigrants [33], limited research has been done to explore how English language proficiency impacts HPV vaccine uptake among foreign-born men and women in the U.S. It is crucial to examine associations between English proficiency and HPV vaccination uptake in foreign-born men and women to tailor interventions better to ameliorate the impact of language-related barriers to HPV vaccine uptake.

This study aims to investigate the association between English proficiency and the uptake of the HPV vaccine among foreign-born individuals, using data from the NHIS survey. We hypothesize that foreign-born individuals with low English proficiency will be less likely to have received the HPV vaccination.

## Methods

### Study design and setting

This is a cross-sectional analysis of data from the National Health Interview Survey (NHIS) dataset. The NHIS data is the largest national survey conducted by the Centers for Disease Control and Prevention’s National Center for Health Statistics. This publicly available, nationally representative data monitors the health of the civilian, noninstitutionalized population within the United States by collecting yearly data on various health topics. The data are collected through face-to-face interviews conducted in participants’ homes and include demographic, socioeconomic, and health-related information. Participant follow-ups may be conducted over the telephone or when a personal visit is difficult to schedule [34]. In the current study, we utilized data collected in 2015, 2016, 2017, and 2018. The decision to use the 2015–2018 dataset was made to ensure an adequate sample size, as 2018 was the last year data on English language proficiency was collected and operationalized in a manner relevant to this study.

### Study population

**Fig. 1:**
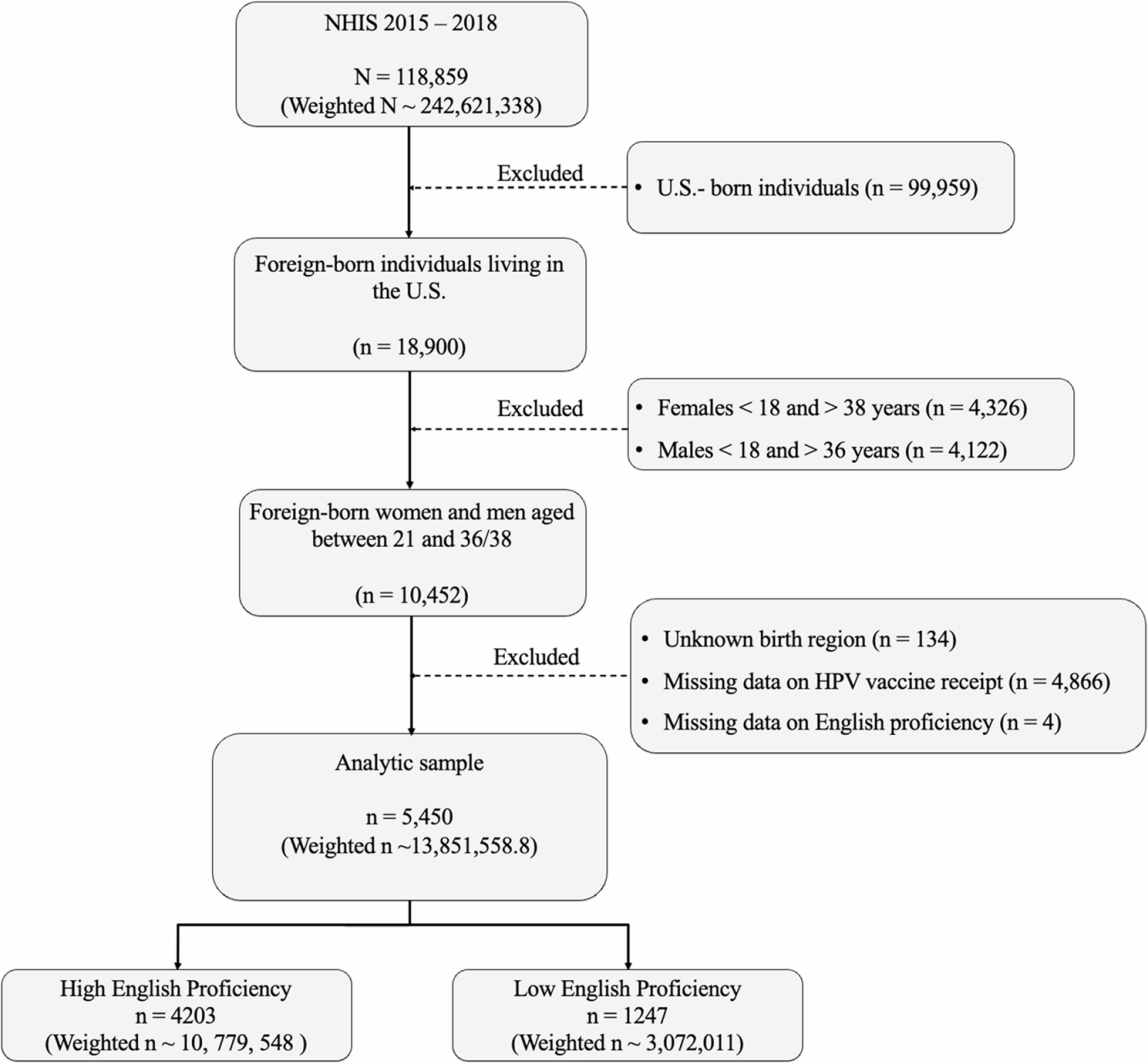
Flow diagram depicting exclusion criteria and final analytic sample of foreign-born U.S adults from the National Health Interview Survey (NHIS), 2015–2018)

The combined dataset (2015–2018) included 118,859 respondents. We excluded individuals born in the United States (*n* = 99,959), resulting in 18,900 foreign-born adults. We then applied age restrictions, retaining foreign-born women aged 18–38 years and men aged 18–36 years (*n* = 10,452), corresponding to groups eligible for HPV vaccination based on vaccine approval timelines (2006 for women, 2009 for men) [35]. Individuals with unknown region of birth (*n* = 134), missing data on HPV vaccine receipt (*n* = 4,866), or missing English proficiency data (*n* = 4) were excluded. The final analytic sample included 5,450 respondents (weighted *N* ≈ 13.85 million) (Fig. 1).

### Measures

The dependent variable was the respondents’ response to the question, “Have you ever received an HPV shot or vaccine?” Responses were dichotomized (yes and no).

The independent variable was the respondent’s response to the question, “How well do you speak English?” The responses were 1) very well, 2) well, 3) not well, and 4) not at all. We classified participants as having high proficiency if they responded, ‘very well’ or ‘well’, and as having low English proficiency if they responded, ‘not well’ or ‘not at all’.

Covariates included gender dichotomized as (male and female), age, years spent in the U.S. recoded to (less than five years, five to less than ten years, and ten years or more), U.S. citizenship status categorized as (citizen and non-citizen), and marital status categorized as (married/cohabiting and not married). The respondents’ socioeconomic status was assessed using employment status categorized as (working for pay, job but not at work, looking for work, unpaid family work and not working/not looking), educational attainment renamed and recoded into (less than high school, high school/GED, some college/associate’s/bachelor’s degree and graduate degree), and household income level categorized as ($0-$34,999, $35,000-$74,999, $75,000-$99,999 and $100,000 and over). Respondents’ access to health care was assessed by the availability of a place to go when sick or in need of health advice, dichotomized as (yes and no), and insurance status was dichotomized as ‘had insurance at the time of the survey’ and ‘uninsured at the time of the survey’.

### Statistical analysis

**Fig. 2:**
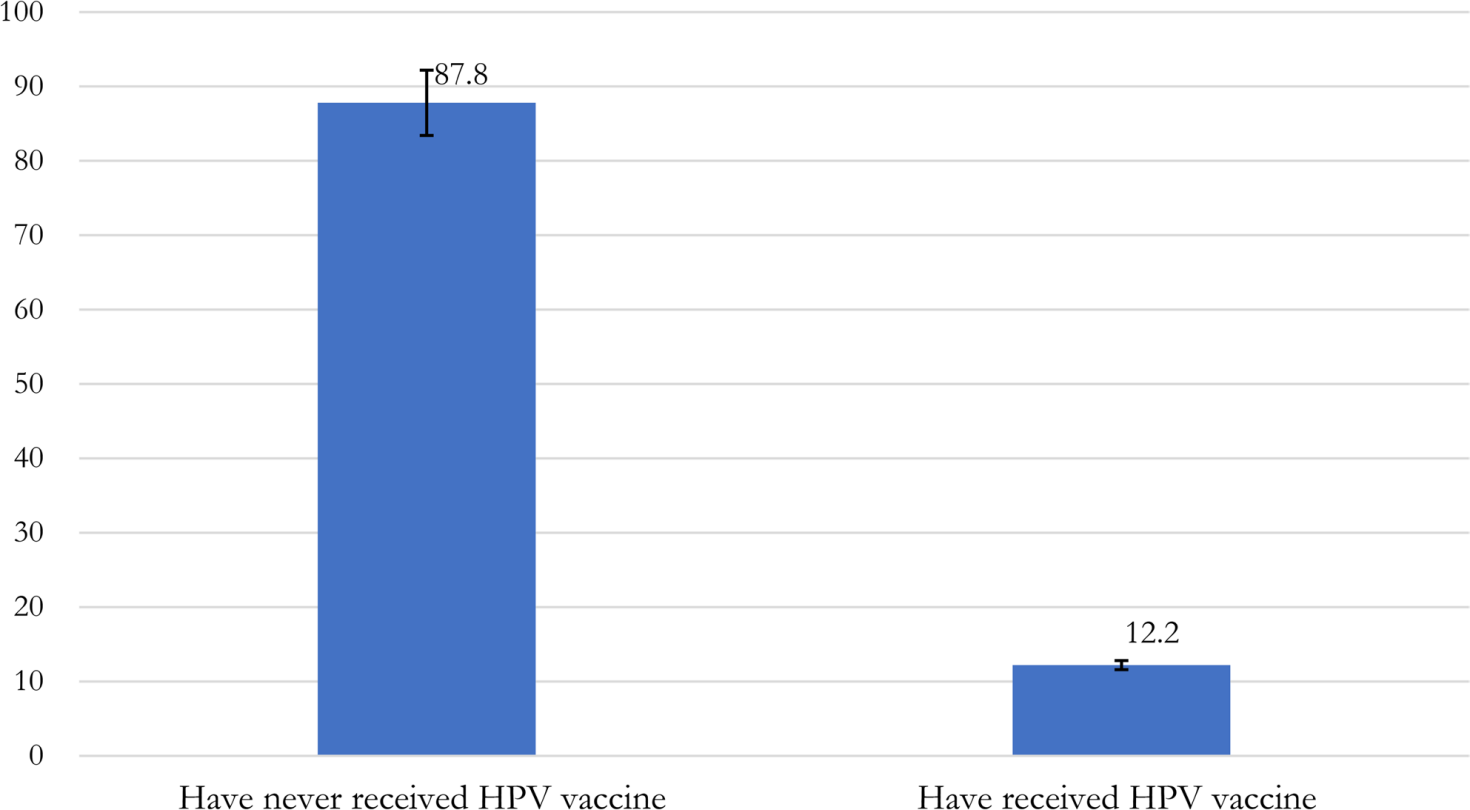
Prevalence of Human Papillomavirus vaccination among foreign-born men and women, NHIS 2015–2018

All analyses were conducted using the Statistical Analysis System (SAS) 9.4. package. Descriptive statistics were used to summarize the respondents’ characteristics by HPV vaccination status, and by English proficiency. Chi-square tests were used to determine the relationship between the categorical variables and t-tests were used for continuous variables. The overall prevalence of HPV vaccination was calculated as the proportion of respondents who reported receiving the HPV vaccine dose, divided by the total number of respondents, and multiplied by 100. Weighted estimates were presented in Figure. 2. We estimated the unadjusted and adjusted odds ratios and 95% confidence intervals for the association between English proficiency and receipt of the HPV vaccine using logistic regression. Predicted probabilities and 95% confidence intervals were derived from logistic regression models using average marginal effects for categorical predictors and marginal effects at the means for the continuous variable (age), providing model-adjusted estimates of vaccination likelihood among foreign-born adults. In the multivariable analyses, we adjusted for all covariates that differed by HPV vaccination status in the bivariate analysis (*p* < 0.05), including sex, age, marital status, U.S. citizenship status, region of birth, education, insurance status, and having a usual source of care. Statistical significance was determined at a p-value < 0.05 in the adjusted models. All estimates were weighted to account for the NHIS’s complex survey design and to produce nationally representative results.

## Results

As shown in Table 1, the analytic sample included 5,450 participants (weighted *N* ~ 13,851,559; high English proficiency: *n* = 4,203, *N* ~ 10,779,548; low proficiency: *n* = 1,247, *N* ~ 3,072,011). The weighted prevalence of ever receiving the HPV vaccine was 12.2% [95% CI: 11.1–13.3] among foreign-born adults in the U.S (Figure. 2). Overall, the sample was 46.8% female [95% CI: 45.1–48.6] and 53.2% male [95% CI: 51.4–54.9], with a mean age of 29.4 years [95% CI: 29.2–29.6]. Compared with individuals in the high-proficiency group, those with low English proficiency were older on average (30.8 vs. 29.0 years), had a higher proportion of males (57.2% vs. 52.0%), and lower proportion of U.S. citizens (14.4% vs. 46.5%). Among respondents with low English proficiency, 85.1% (95% CI [82.3, 87.6]) were born in Latin America, the Caribbean, or South America, compared to 46.1% (95% CI [43.4, 48.8]) in the high English proficiency group (Tables 1).

**Table 1:** Characteristics of U.S. foreign-born men and women overall and by english Proficiency, National Health Interview Survey (NHIS), 2015–2018 (Weighted percentages and 95% confidence Intervals)

	Overall	English proficiency		*p*-value
		High	Low	
Unweighted sample	*n* = 5,450	*n* = 4203	*n* = 1247	
Weighted sample	*N* ~ 13,851,558.8	*n* ~ 10, 779, 548	*n* ~ 3,072,011	
Participants’ Characteristics	Weighted % (95 CIs)			
Ever received HPV vaccine				
No	87.8 (0.87–0.89)	85.6 (84.1–86.9)	95.7 (94.2–96.8)	< 0.001
Yes	12.2 (11.1–13.3)	14.4 (13.1–15.9)	4.3 (3.1–5.8)	
Sex				
Male	53.2 (51.4–54.9)	52.0 (50.0–54.0)	57.2 (53.6–60.7)	0.012
Female	46.8 (45.1–48.6)	48.0 (46.0–49.9)	42.8 (39.3–46.4)	
Age, *weighted mean (95% CIs)*	29.4 (29.2–29.6)	29.0 (28.7–29.3)	30.8 (30.4–31.2)	< 0.001
Marital status				
Married or cohabiting	59.5 (57.7–61.3)	55.7 (53.6–57.9)	72.7 (69.4–75.8)	< 0.001
Not married	40.5 (38.7–42.3)	44.2 (42.1–46.4)	27.3 (24.2–30.6)	
Citizenship status				
U.S. Citizen	39.4 (37.5–41.3)	46.5 (44.2–48.8)	14.4 (12.0–17.3)	< 0.001
Non-citizen	60.6 (586–62.5)	53.5 (51.2–55.8)	85.6 (82.7–88.0)	
Household Income				
$0 - $34,999	37.4 (35.4–39.5)	33.6 (31.4–35.9)	51.5 (47.1–55.9)	< 0.001
$35,000 - $74,999	31.6 (29.7–33.6)	31.7 (29.4–34.0)	31.4 (27.7–35.3)	
$75,000 - $99,999	10.7 (9.3–12.2)	11.6 (10.0–13.5)	7.2 (5.1–10.2)	
$100,000 and over	20.3 (18.5–22.3)	23.1 (21.0–25.4)	9.9 (7.2–13.3)	
Employment				
Working for pay	68.0 (66.2–69.8)	70.1 (68.0–72.2)	60.6 (57.1–64.0)	< 0.001
Not working/not looking	24.4 (22.7–26.1)	22.0 (20.1–24.0)	32.8 (29.5–36.2)	
Job but not at work	1.8 (1.4–2.3)	2.0 (1.6–2.6)	1.2 (0.5–2.3)	
Looking for work	5.3 (4.5–6.3)	5.5 (4.5–6.8)	4.8 (3.5–6.5)	
Unpaid family work	0.4 (0.2–0.8)	0.4 (0.2–0.9)	0.7 (0.2–2.3)	
Educational attainment				
Less than high school	20.1 (18.5–21.8)	10.4 (9.0–11.9)	54.7 (50.9–58.3)	< 0.001
High school/GED*	21.8 (20.2–23.6)	21.0 (19.2–23.0)	24.8 (21.7–28.2)	
Some college/Associate’s/Bachelor’s degree	45.2 (43.3–47.0)	52.7 (50.7–54.8)	18.3 (15.7–21.2)	
Graduate degree or above	12.9 (11.5–14.4)	15.9 (14.3–17.7)	2.2 (1.5–3.4)	
Geographical Region of Birth				
Latin America/Caribbean/South America	54.7 (52.2–57.2)	46.1 (43.4–48.8)	85.1 (82.3–87.6)	< 0.001
Europe & Russia	9.1 (8.2–10.2)	11.5 (10.3–12.9)	0.9 (0.5–1.9)	
Africa	6.8 (5.7–8.1)	8.1 (6.8–9.7)	2.1 (1.3–3.2)	
Middle East	3.0 (2.4–3.7)	3.3 (2.6–4.1)	1.7 (1.0–3.0)	
Indian Subcontinent	10.6 (9.3–12.1)	12.9 (11.3–14.6)	2.6 (1.6–4.1)	
Asia and Southeast Asia	13.0 (11.8–14.4)	14.7 (13.3–16.3)	7.3 (5.6–9.5)	
Elsewhere	2.6 (2.0–3.3)	3.3 (2.6–4.2)	0.2 (< 0.01 −1.0)	
Years lived in U.S.				
Less than 5 years	21.0 (19.4–22.8)	19.1 (17.3–21.0)	27.9 (24.5–31.6)	< 0.001
Between 5 and less than 10 years	17.2 (15.8–18.7)	16.1 (14.6–17.7)	20.9 (17.8–24.4)	
Greater or equal to 10 years	61.8 (59.8–63.8)	64.8 (62.6–66.9)	51.2 (47.2–55.2)	
Insurance status at time of survey				
Insured	71.3 (69.2–73.2)	79.4 (77.4–81.3)	42.7 (39.1–46.5)	< 0.001
Uninsured	28.7 (26.8–30.8)	20.6 (18.7–22.6)	57.3 (53.4–60.9)	
Has place to go when sick/needs health advice?				
No	30.7 (28.8–32.5)	27.3 (25.4–29.3)	42.4 (38.5–46.3)	< 0.001
Yes	69.3 (67.5–71.2)	72.7 (70.7–74.6)	57.6 (53.7–61.5)

**Table 2:** Characteristics of U.S. foreign-born men and women by human papillomavirus (HPV) vaccine Receipt, National Health Interview Survey (NHIS), 2015–2018 (Weighted percentages and 95% confidence Intervals)

	Ever received HPV shot/vaccine		*p*-value
	No	Yes	
Unweighted sample	*n* = 4766	*n* = 684	
Weighted sample	*n* ~ 12, 165, 926	*n* ~ 1, 685, 632	
Participants’ Characteristics	Weighted % (95% CIs)		
Sex			
Male	50.1 (48.2–52.0)	23.3 (19.3–27.8)	< 0.001
Female	49.9 (48.0–51.8)	76.7 (72.2–80.7)	
Age, *weighted mean (95% CIs)*	29.8 (29.6–30.1)	26.2 (25.6–26.9)	< 0.001
Marital status			
Married or cohabiting	62.4 (60.6–64.2)	38.7 (34.2–43.5)	< 0.001
Not married	37.8 (35.8–39.4)	61.3 (56.5–65.8)	
Citizenship status			
U.S. Citizen	37.8 (35.8–39.8)	51.0 (46.0–56.0)	< 0.001
Non-citizen	62.2 (60.2–64.2)	49.0 (44.0–54.9)	
Household Income			
$0 - $34,999	37.6 (35.4–39.9)	36.1 (30.7–41.9)	0.570
$35,000 - $74,999	31.5 (29.4–33.6)	32.5 (27.3–38.1)	
$75,000 - $99,999	10.9 (9.4–12.6)	8.9 (6.1–12.9)	
$100,000 and over	20.0 (18.1–22.0)	22.5 (18.2–27.5)	
Employment			
Working for pay	68.6 (66.5–70.5)	64.0 (59.1–68.6)	0.128
Not working/not looking	1.9 (1.5–2.4)	1.5 (0.8–2.3)	
Job but not at work	0.5 (4.2–6.1)	7.4 (4.7–11.3)	
Looking for work	0.3 (0.2–9.0)	0.9 (0.3–2.3)	
Unpaid family work	24.1 (22.3–26.0)	26.2 (22.1–30.9)	
Educational attainment			
Less than high school	21.4 (19.6–23.2)	11.0 (8.0–14.8)	< 0.001
High school/GED*	22.6 (20.8–24.4)	16.6 (13.1–20.9)	
Some college/Associate’s/Bachelor’s degree	43.3 (41.3–45.2)	58.8 (53.7–63.7)	
Graduate degree or above	12.8 (11.4–14.3)	13.6 (10.6–17.3)	
Geographical Region of Birth			
Latin America/Caribbean/South America	55.7 (53.0–58.3)	48.0 (42.9–53.3)	< 0.001
Europe & Russia	8.6 (7.6–9.7)	13.2 (10.3–16.7)	
Africa	6.6 (5.5–7.9)	8.3 (5.9–11.6)	
Middle East	3.1 (2.5–3.9)	1.9 (1.0–3.5)	
Indian Subcontinent	11.3 (9.9–12.8)	5.9 (3.9–9.0)	
Asia and Southeast Asia	12.3 (11.0–13.8)	18.6 (15.1–22.6)	
Elsewhere	2.4 (1.9–3.1)	4.0 (2.6–6.2)	
Years lived in U.S.			
Less than 5 years	21.4 (19.6–23.4)	18.3 (15.1–22.0)	0.218
Between 5 and less than 10 years	17.3 (15.8–18.9)	16.1 (12.7–20.2)	
Greater or equal to 10 years	61.3 (59.1–63.4)	65.6 (61.1–69.9)	
Insurance status at time of survey			
Insured	69.3 (67.1–71.4)	85.4 (81.4–88.6)	< 0.001
Uninsured	30.7 (28.6–32.9)	14.6 (28.6–32.9)	
Has place to go when sick/needs health advice?			
No	32.1 (30.1–34.1)	20.6 (16.9–24.8)	< 0.001
Yes	67.9 (65.9–69.9)	79.4 (75.2–83.1)	

Among those who had not received the HPV vaccine, 49.9% (95% CI [48.0, 51.8]) were female and 50.1% (95% CI [48.2, 52.0]) were male (Table 2). In contrast, among vaccinated respondents, 76.7% (95% CI [72.2, 80.7]) were female and 23.3% (95% CI [48.0, 51.8]) were male (*p* < 0.001). Unvaccinated respondents were also older on average (29.8 years, 95% CI [29.6, 30.1]) compared to vaccinated respondents (26.2 years, 95% CI [25.6, 26.9]; *p* < 0.001). Regional patterns also differed between groups: 55.7% (95% CI [53.0, 58.3]) of unvaccinated respondents were born in Latin America, the Caribbean, or South America, and 12.3% (95% CI [11.0, 13.8]) were born in Asia or Southeast Asia (Table 2). In comparison, among vaccinated respondents, 48.0% (95% CI [42.9, 53.3]) were born in Latin America, the Caribbean, or South America, and 18.6% (95% CI [15.1, 22.6]) were born in Asia or Southeast Asia. 67% (67.9%; 95% CI [65.9, 69.9]) of unvaccinated respondents reported having a usual source of care, compared to 79.4% (95% CI [75.2, 83.1]) of vaccinated respondents (*p* < 0.001) (Table 2).

In the adjusted model, foreign-born individuals who reported low English proficiency had 52% lower odds of receiving the HPV vaccine than those with high English proficiency (OR = 0.48; 95% CI: 0.32–0.73). The predicted probability of HPV vaccination was 8.78% [95% CI: 7.91–9.64] among those with high English proficiency, compared to 4.92% [3.35–6.49] among those with low proficiency (Table 3). 

**Table 3:** Logistic regression of the association between human papillomavirus (HPV) vaccination and english Proficiency, National Health Interview Survey (NHIS), 2015–2018: adjusted odds ratios and predicted probabilities (95% confidence Intervals)

	Adjusted Odds Ratios (95% CI)	Predicted probabilities (95% CI)
English proficiency		
High	**ref**	8.78 (7.91–9.64)
Low	0.48 (0.32–0.73)*	4.92 (3.35–6.49)
Sex		
Male	ref	3.90 (3.12–4.68)
Female	4.19 (3.19–5.50)*	12.33 (11.09–13.58)
Age, *weighted mean (95% CIs)*	0.88 (0.86–0.91)*	11.50 (10.20–12.71)^##^
Marital status^*#*^		
Married or cohabiting	ref	6.83 (5.85–7.80)
Not married	1.55 (1.20–2.00)*	9.60 (8.35–10.81)
Citizenship status		
U.S. Citizen	ref	9.02 (7.86–10.19)
Non-citizen	0.78 (0.61–1.00)	7.49 (6.52–8.46)
Educational attainment^*#*^		
Less than high school	ref	6.96 (4.93–8.99)
High school/GED*	0.88 (0.57–1.38)	6.31 (4.85–7.78)
Some college/Associate’s/bachelor’s degree	1.31 (0.86–2.00)	8.50 (7.56–9.54)
Graduate degree or above	2.07 (1.23–3.49)*	11.8 (9.20–14.47)
Geographical Region of Birth		
Latin America/Caribbean/South America	ref	8.68 (7.50–9.85)
Europe & Russia	1.13 (0.78–1.64)	9.50 (7.28–11.72)
Africa	1.05 (0.65–1.68)	8.97 (6.03–11.91)
Middle East	0.47 (0.24–0.92)	4.72 (2.11–7.34)
Indian Subcontinent	0.39 (0.23–0.68)*	4.10 (2.37–5.82)
Asia and Southeast Asia	1.05 (0.75–1.46)	9.00 (7.27–10.73)
Elsewhere	1.40 (0.80–2.43)	11.05 (7.12–14.99)
Insurance status at time of survey^#^		
Insured	ref	8.72 (7.84–9.59)
Uninsured	0.64 (0.45–0.91)*	6.19 (4.65–7.72)
Has usual source of care^*#*^		
No	ref	7.09 (5.73–8.45)
Yes	1.28 (0.97–1.69)	8.56 (7.69–9.42)
The study sample included foreign-born adults aged 18–38 years (women) and 18–36 years (men) who participated in the 2015–2018 National Health Interview Survey (NHIS). Individuals were required to have complete data on HPV vaccination status, English proficiency, and region of birth		

#Missing data were as follows: marital status (*n* = 6), insurance status (*n* = 20), education (*n* = 19), usual source of care (*n* = 3), and citizenship status (*n* = 47). The complete case analysis included 5,355 respondents. In the unadjusted (null) model, foreign-born individuals with low English proficiency had significantly lower odds of receiving the HPV vaccine compared to those with high proficiency (OR = 0.27; 95% CI [0.19, 0.37]). Corresponding predicted probabilities were 14.4% (95% CI [13.0%, 15.8%]) for those with high English proficiency and 4.3% (95% CI [3.0%, 5.6%]) for those with low English proficiency.

^##^Predicted probabilities at sample mean age 29.4

*Significant at* p < 0.05*

The model accounted for sex, age, marital status, U.S. citizenship status, region of birth, education, insurance status, and having a usual place to go for health needs. Predicted vaccination probability was higher among women (12.33% [11.09–13.58]) compared to men (3.90% [3.12–4.68]). At the mean age of 29.4 years, the predicted probability of ever having received the HPV vaccine was11.5% (95% CI: 10.2%–12.7%). Those who were not married had a higher predicted probability (9.6% [8.35–10.81]) compared to those who were married or cohabiting (6.83% [5.85–7.80]). Individuals with a graduate degree or higher had the highest predicted probability (11.8% [9.20–14.47]) compared to those with less education. By region of birth, predicted vaccination probability was lowest among those born in the Indian Subcontinent (4.10% [2.37–5.82]) and the Middle East (4.72% [2.11–7.34]), and highest among those born in Europe & Russia (9.50% [7.28–11.72]) and Elsewhere (11.05% [7.12–14.99]). Those who were insured had a higher predicted probability (8.72% [7.84–9.59]) compared to the uninsured (6.19% [4.65–7.72]) Predicted probabilities of HPV vaccination were 9.0% (95% CI [7.9%, 10.2%]) among U.S. citizens and 7.5% (95% CI [6.5%, 8.5%]) among non-citizens; however, this difference was not statistically significant. (Table 3).

## Discussion

This study examined the relationship between English language proficiency and HPV vaccine uptake among foreign-born individuals in the U.S., using data from the NHIS (2015–2018). Findings show that low English proficiency was significantly associated with 52% lower odds of HPV vaccine receipt among foreign-born individuals, which aligns with our hypothesis that those who report low English proficiency are less likely to have received the HPV vaccine. The current study findings also show an overall low HPV vaccine prevalence (12.2%) among the foreign-born population, which also aligns with previous studies that report suboptimal HPV vaccine rates [14, 36]. The low HPV vaccine receipt among this population places them at increased risk for HPV infections and associated cancers, considering the higher burden of HPV and HPV-related cancers in regions such as Latin America, Africa, parts of Europe (including Romania, Bulgaria, Ukraine), and Asia [37]. The recent expansion of eligibility for the HPV vaccine to individuals aged 27 to 45 years in the U.S. presents an opportunity for foreign-born adults to catch up on vaccination, which could help reduce HPV vaccination disparities in this group.

Few studies have assessed the relationship between English proficiency and HPV vaccine uptake and knowledge among specific immigrant population groups [38–40]. These studies focused on Vietnamese Americans, Arab Americans, and people of Indian Ancestry, and found that higher English proficiency was associated with increased likelihood of HPV vaccine receipt and awareness. The current study expands their work to assess the association in foreign-born individuals using a national sample, and our findings are consistent with theirs. Low English proficiency is a social determinant of health-related factors that significantly impacts access to care, quality of care, and ultimately, health outcomes [41–43]. This is particularly true of the U.S. healthcare system, which is complex and challenging for individuals who are not proficient in the dominant language used within it [44]. Even more so if seeking HPV vaccination when older, as it will require effective communication with providers and multiple interactions within the healthcare system to access services [45]. Limited English proficiency creates language barriers that reduce knowledge and utilization of preventive services such as the HPV vaccine [46]. This diminished awareness may partly explain the lower HPV vaccination rates observed among foreign-born populations.

Our findings indicate several factors associated with a lower probability of receiving HPV vaccination, including male sex, older age, lack of insurance at the time of the survey, and lack of a usual source of care. The observed sex differences are consistent with prior studies and are likely influenced by the temporal and sex-specific nature of HPV vaccine recommendations [47]. The vaccine was initially recommended for females in 2006, with male recommendations introduced later in 2009 (permissive) and 2011 (routine), accompanied by targeted marketing and cervical cancer–focused messaging [48–50]. This historical emphasis on females likely contributed to lower perceived vaccine relevance among males, resulting in persistent sex-based disparities. Similarly, age-based differences may reflect these temporal factors, with older adults being less likely to have been eligible for vaccination during the early years of vaccine introduction [48–50]. Thus, having fewer opportunities for routine vaccination. Factors such as lack of insurance and lack of a usual source of care are upstream determinants that limit access to preventive services, particularly for immigrants who are often excluded from federal and state public insurance programs [51]. 

Our results highlight the disparities in HPV vaccine uptake among foreign-born individuals with low English proficiency. Trained medical interpreters represent an evidence-based strategy to strengthen patient–provider communication and improve care for populations with low English proficiency, but challenges related to cost, access, staffing shortages, and accuracy remain important considerations when implementing this approach in care settings [52–54]. Additionally, integrating curriculum-based training on the use of medical interpreters and communication with low-English-proficient patients can equip medical students to engage with linguistically diverse populations in clinical practice more effectively [55].

### Implications for practice and research

We highlight two important implications. In practice, our findings suggest the need to implement and expand programs within the health care systems aimed at providing linguistically appropriate educational materials and services to increase the knowledge and uptake of the HPV vaccine among foreign-born individuals [56]. The development of such materials should involve the target population to ensure better comprehension of the information and the incorporation of the cultural beliefs and values of the subpopulation [57]. Improving or expanding interpreter services, while addressing barriers to their use such as cost, can help close communication gaps and reduce HPV vaccination disparities in foreign-born patients with limited English proficiency [58]. 

Implications for research include the need for prospective studies to explore the associations between English proficiency among Foreign-born populations, incorporating other measures of English proficiency that can better capture and expand upon our understanding of its impact on HPV vaccine uptake [59]. This includes concepts like foreign language anxiety, which has been extensively researched in the field of education [60], but has not been explored in the context of preventive services utilization among low-English-proficient individuals. Research should also explore the role of artificial intelligence interpreter service use within healthcare settings to improve patient-provider communication about HPV and HPV vaccination [61].

### Limitations

This study has limitations. First, we used data from 2015 to 2018, which may not accurately reflect current trends in English proficiency or HPV vaccination receipt among the population of interest. The analysis was restricted to data from 2015 to 2018, as the variables of interest were last collected in 2018. Using older data is unlikely to bias study estimates, as the association between English proficiency and HPV vaccination is not expected to change substantially over time. Second, we used self-reported measures of English proficiency and HPV vaccination. Considering the limited awareness of HPV vaccination in this population [62], this might impact self-report of ever receiving the vaccine. Additionally, the interval between HPV vaccine receipt and the time of the survey data collection carries a potential for recall bias. Recall bias and limited awareness may lead to either underreporting or overreporting of HPV vaccination. For example, some individuals may have received the vaccine but forgotten, while others may mistake another vaccine for the HPV vaccine. This non-differential misclassification will likely bias our estimates towards the null, meaning the true association between English proficiency and HPV vaccination may be stronger than what we observed. Third, we did not consider the number of doses respondents received; therefore, we cannot determine if those who reported receiving the vaccine received all doses. Fourth, excluding respondents with missing data on HPV vaccination status may have introduced bias, as this group represented approximately 9% of foreign-born adults who were eligible for inclusion in the analytic sample. Last, this is a cross-sectional study and does not assess the temporal effects between English proficiency and HPV vaccine receipt.

## Conclusion

Our findings suggest that limited English proficiency negatively influences receipt of HPV vaccination among foreign-born individuals in the U.S. Language barrier significantly impacts access and health outcomes, particularly among foreign-born populations. Targeted interventions for populations in need of linguistically tailored education and interactions within healthcare systems are necessary to address disparities in HPV vaccination and promote health equity.

## Data Availability

The data analyzed in the current study are publicly available on the CDC’s NCHS website at https://archive.cdc.gov/www_cdc_gov/nchs/nhis/1997-2018.htm.

## Abbreviations

HPV: Human Papillomavirus
U.S.: United States
NHIS: National Health Interview Survey
OR: Odds Ratio
CI: Confidence interval
